# Prenatal exposure to di(2-ethylhexyl) phthalate alters the association of glutamatergic proteins with PTEN in the hippocampus of male rat offspring

**DOI:** 10.1016/j.ibneur.2025.12.005

**Published:** 2025-12-13

**Authors:** Natalia Kiknadze, Elene Zhuravliova, David Mikeladze

**Affiliations:** a Institute of Chemical Biology, Ilia State University, 3/5 Cholokashvili av, Tbilisi 0179, Georgia; b I.Beritashvili Center of Experimental Biomedicine, 14 Gotua St, Tbilisi 0162, Georgia

**Keywords:** DEHP, Hippocampus, Glutamate, Prenatal exposure, PTEN

## Abstract

Phthalates are extensively used chemicals known to have adverse effects on human health. Prenatal exposure to phthalates has been associated with potential disruptions in brain development and an elevated susceptibility to cognitive and behavioral disorders. The effects of phthalates on learning, memory, and related hippocampal processes have been widely studied; however, the molecular pathways through which phthalates modulate synaptic processes are not fully understood. Previous studies have shown that the molecular mechanism of DEHP-induced hippocampal neurotoxicity in the maturing male brain involves changes in phosphatase and tensin homolog (PTEN) subcellular location, which suppresses Akt/mTOR signaling and enhances GluN2B NMDA-mediated synapse depression. Immunoprecipitation experiments revealed that the prenatal administration of DEHP to rats led to a reduction in the association of the scaffold protein NHERF1, NMDA receptor subunits, AMPA receptor subunits, metabotropic glutamate receptor 5, and excitatory amino acid transporter-2 with PTEN in the hippocampus of offspring, while the overall quantity of these proteins remained unchanged. Furthermore, our results demonstrated that prenatal exposure of rats to phthalates resulted in downregulation of calcineurin phosphatase activity, decreased autophosphorylation of calcium/calmodulin-dependent protein kinase II, reduced protein kinase A activity, and upregulation of Akt kinase in the hippocampus of young rats. These findings suggest that the susceptibility of the PTEN protein interactome to phthalates in the glutamatergic postsynaptic density may influence synaptic plasticity at excitatory neurons in the hippocampus of offspring after exposure of parent rats to DEHP during gestation.

## 1 Introduction

Phthalates, widely employed as plasticizers in industrial manufacturing to impart elasticity to plastic products, have attracted considerable attention regarding their impact on human health (Cook and Halden, 2020). The pervasive use of phthalates and the potential for human exposure have prompted extensive scholarly investigations into their health implications (Sree et al., 2023, Wang and Qian, 2021). The focus of scientific inquiry has mainly been on prenatal exposure to phthalates, with specific studies establishing associations between maternal exposure to certain phthalates and adverse birth outcomes (Lucaccioni et al., 2021). Given their capacity to traverse the placental barrier, phthalates pose a potential risk of exposure during fetal development (Natividade et al., 2023). Phthalates can impact various organs and systems within the human body (Lucaccioni et al., 2021) among which is CNS. The biochemical actions of phthalates are intricate and involve diverse mechanisms. Certain phthalates interfere with hormone receptors, potentially disrupting normal endocrine function and impacting reproductive and developmental processes through antiandrogenic, androgenic, and estrogenic activity (Lupu et al., 2023).

Over the past years, research on the neurotoxic effects of various phthalates has increased. Building on the evidence of phthalates’ ability to cross the BBB or placental barrier and affect multiple organ systems, recent epidemiological, animal, and mechanistic studies have converged on their neurodevelopmental toxicity (Hlisníková et al., 2021). Phthalates, both high- and low-molecular-weight compounds—particularly di(2-ethylhexyl) phthalate (DEHP), di-n-butyl phthalate (DBP), diisononyl phthalate (DINP), and butyl benzyl phthalate (BBP)— even at background exposure levels, have been linked to developmental impairments, behavioral disorders, improper neural signaling, impaired special learning and memory, etc. (Huang et al., 2022, Ma et al., 2015, Safarpour et al., 2022, Silano et al., 2019). At the cellular level, DEHP’s primary metabolite, mono(2-ethylhexyl) phthalate (MEHP), blocks androgen receptors, activates peroxisome proliferator-activated receptors, and causes oxidative stress, mitochondrial dysfunction, and dopaminergic neuron loss in rodent models (Huang et al., 2022) In contrast, DBP and BBP induce microglial activation, increase proinflammatory cytokines, and compromise blood–brain barrier integrity in vitro. Epigenomic assays further show changes in DNA methylation of neurotrophic genes, connecting phthalate exposure to lasting modifications in neuronal growth, connectivity, and synaptic plasticity (Lucaccioni et al., 2021, Maric et al., 2024, Tseng et al., 2014).

The effects of phthalates on learning, memory, and related hippocampal processes have been thoroughly researched (Kougias et al., 2018, Lv et al., 2022, Safarpour et al., 2021). Based on previous studies on phthalate neurotoxicity and their impact on neural plasticity, it appears that phthalates cause disruptions in hippocampal structure and plasticity during prenatal, perinatal, and neonatal development (Holahan and Smith, 2015). *In vitro* studies suggest that phthalates can induce burst firing in hippocampal neurons, leading to neurotoxicity (Matsunaga et al., 2010). Meanwhile, *in vivo* studies demonstrate that prenatal exposure to DEHP results in deficits in spatial learning and memory retention in offspring (Xu et al., 2015). Exposure to phthalates is thought to alter cognitive and behavioral traits, leading to lower IQ, attention deficits, hyperactivity, and impaired social communication in children (Arbuckle et al., 2016). Chronic exposure of adult male mice to environmentally relevant doses of DEHP affects hippocampal function and structure, as well as alters behavioral patterns (Ducroq et al., 2023). The biochemical mechanisms by which phthalates affect nerve cells involve diverse pathways, and ongoing epidemiological research continues to explore their potential impact on neurobehavioral development, including possible links to attention deficit hyperactivity disorder (ADHD) and other cognitive and behavioral issues (Mankidy et al., 2013). Emerging evidence suggests that phthalates may impact mitochondrial function, and disruptions in this area can have far-reaching consequences for cellular processes, potentially contributing to adverse health outcomes (Duarte-Hospital et al., 2021). It is important to emphasize that the specific biochemical actions of different phthalates may vary, and their effects depend on factors such as the type of phthalate, dosage, duration of exposure, and individual susceptibility. Therefore, the direct causal relationship and mechanisms linking phthalates to neurodevelopment and neurotoxicity have not yet been firmly established, and the molecular pathways involved in phthalates' effects on synaptic processes remain incompletely understood (Engel et al., 2021).

Besides epigenetic remodeling, phthalate exposure disrupts the PTEN/Akt pathway, weakening essential survival signals in developing neurons. DEHP-activated PPARγ signaling suppresses PTEN transcription, while simultaneous oxidative stress causes PTEN oxidation and inactivation. The Phosphatase and Tensin homolog (PTEN) is a critical tumor suppressor that functions as a dual-specificity phosphatase, primarily regulating the Phosphatidylinositol 3-Kinase/Akt (PI3K/Akt) signaling pathway. PTEN acts by converting the second messenger PIP3 (phosphatidylinositol (3,4,5)-trisphosphate) back to PIP2 (phosphatidylinositol (4,5)-bisphosphate), thereby antagonizing the activation of Akt. Since the PI3K/Akt pathway is a central regulator of cellular processes, including proliferation, survival, metabolism, and motility, PTEN's role is essential for maintaining cell homeostasis. Dysregulation of PTEN is implicated in numerous pathologies, including cancer, metabolic disorders, and neurodevelopmental and neuropsychiatric disorders (Lu et al., 2023) As a result, PI3K/Akt pathways become overactive, leading to cell cycle arrest and apoptosis in neural cells. This mechanistic convergence—endocrine disruption, chromatin modification of neurotrophic loci, and PTEN loss—directly links phthalate exposure to impaired neurodevelopmental outcomes (Hlisníková et al., 2021).

Our previous study revealed that prenatal exposure to DEHP at certain doses causes permanent changes in the male offspring’s hippocampus, resulting in impaired cognition and elevated anxiety. This effect is linked to altered PTEN localization, which disrupts Akt/mTOR signaling, increases GluN2B-mediated synaptic depression, and reduces mitochondrial fusion (Kiknadze et al., 2025).

This molecular disruption of key regulatory and synaptic proteins highlights a mechanism of environmental toxicity that is directly related to the regulation of protein complexes. In recent years, protein-protein interactions (PPIs) have received increasing attention and have become attractive targets for drugs and environmental toxins. The identification of this specific signaling cascade strongly suggests the involvement of altered synaptic protein complexes, which are critical drivers of glutamatergic postsynaptic malfunction (Lu et al., 2020). Therefore, this study utilizes proteomics approaches to characterize the PPIs within the DEHP-affected hippocampus, seeking novel therapeutic targets to address the neurobehavioral deficits. Proteomics approaches have identified and characterized multiple synaptic protein complexes in glutamatergic postsynaptic malfunction, which can lead to aberrant signaling in the brain (Baucum, 2017). Dysregulation of PPIs in neurons has been associated with various neurological disorders, including Alzheimer's disease, Parkinson's disease, and Huntington's disease (van Gelder and Altelaar, 2021). Understanding the assembly and disorganization of PPI in neurons is crucial for unraveling the molecular basis of neurological function and developing potential therapeutic strategies for neurological disorders.

Proteins distributed in the proteomic composition within the glutamatergic post-synaptic region drive synaptic plasticity (Baucum, 2017). While core synaptic scaffolds and receptors are ubiquitously present at most excitatory synapses, numerous proteins exhibit distinct expression patterns across various brain regions (Zhu et al., 2018). These proteins congregate to form a protein heterocomplex, occasionally reaching huge molecular weights (Frank et al., 2016). Alterations in this protein interactome can modulate known and novel functions of various regulatory proteins by changing the enzyme activity, protein stability, and subcellular localization (Lautz et al., 2021, Mosessian and Wu, 2010).

Proteins that regulate central biochemical processes in the cell include PTEN (phosphatase and tensin homolog). PTEN is a classical tumor suppressor that antagonizes phosphatidylinositol 3-phosphate kinase (PI3K)/Akt signaling. Although there is a strong association of PTEN germline mutations with cancer syndromes, they have also been described in a subset of patients with autism spectrum disorders characterized by impairments in social interactions and communication, repetitive behavior, and, occasionally, epilepsy (Rademacher and Eickholt, 2019). PTEN regulates both the density and strength of glutamatergic synapses. PTEN and its signal cascade regulate brain development during cell proliferation and differentiation, migration, neurite outgrowth, synaptogenesis, and myelination. (Skelton et al., 2020). PTEN interacts with various proteins, and these interactors are responsible for some functional roles of PTEN beyond the negative regulation of the PI3K pathway (Smith et al., 2020). In the various signaling systems, PTEN forms a heterocomplex with a huge molecular weight (> 650 kDa) involving diverse regulatory proteins (Mosessian and Wu, 2010). Changes in PPI involving PTEN have been shown to play a significant role in the biological mechanism behind many human diseases, which are promising targets for discovering and developing new drugs (Ryan and Matthews, 2005). Based on these considerations, our studies to elucidate the mechanisms of prenatal phthalate exposure have focused on the heterocomplex of proteins formed by PTEN in the hippocampus of offspring. In this study, we found that prenatal exposure of rats to DEHP causes changes in the association of glutamatergic postsynaptic proteins with PTEN, resulting in the modulation of downstream regulatory systems in the hippocampus of offspring. We propose that the susceptibility of the PTEN protein interactome to phthalates in glutamatergic postsynapses may influence synaptic plasticity at excitatory neurons in the hippocampus of offspring after exposure of parent rats to DEHP during gestation.

## 2 Materials and methods

### 2.1 Research permission for ethics

Animal care during experimental procedures was carried out following the recommendations of the Ilia State University Research Projects Ethics Commission (decision letter R/294–23) and the Council of Europe Directive 2010/63 / EU on the protection of animals used for scientific purposes.

### 2.2 Prenatal exposure

Adult male (350–400 g) and female (200–250 g) Wistar rats were obtained from the breeding colony at the I. Beritashvili Center of Experimental Biomedicine (Tbilisi, Georgia). All animals underwent a forced swimming test to exclude outliers displaying extreme behavioral phenotypes that might reflect underlying physiological or neuropsychiatric abnormalities. Rats were housed in groups under controlled temperature conditions (22 ± 2°C) with a 12-hour light/dark cycle, and provided ad libitum access to standard chow and water.

To assess the effects of prenatal gastrointestinal exposure to di(2-ethylhexyl) phthalate (DEHP), selected females were administered 500 mg/kg/day of DEHP dissolved in drinking water. This dosing regimen, based on prior studies (Hsu et al., 2021, Rowdhwal and Chen, 2018, Schmidt et al., 2012) was designed to simulate high-dose human exposure scenarios (e.g., during medical procedures or occupational contact), considering the more rapid metabolism of DEHP in rodents (Dutta et al., 2020). DEHP exposure began three days after pairing, following confirmation of mating. Control females received only drinking water in the same amount.

Pregnancies were confirmed by daily monitoring of body weight. The average gestational length was 20–25 days, with no significant differences between experimental groups. Animals were housed in polycarbonate cages with stainless-steel lids, wood shavings for bedding, and glass water bottles to minimize external phthalate contamination. DEHP administration was discontinued post-delivery to avoid exposure during lactation. By discontinuing dosing before lactation, we avoided confounding postnatal exposure via breast milk—thereby attributing all observed synaptic and PTEN/Akt–mediated alterations specifically to prenatal DEHP exposure.

Each dam produced 10 ± 2 pups on average. Offspring were weaned at postnatal day 21 and housed in same-sex groups of five per cage under identical environmental conditions (20–22°C, 12-h light/dark cycle, standard diet, and water ad libitum). No significant differences in birth weight were observed between groups. However, by ten weeks of age, DEHP-exposed male offspring exhibited a 10 ± 3 % increase in body weight compared to controls.

Only male offspring were included in the subsequent experiments (n = 16 for control; n = 24 for DEHP-exposed). In further experiments, four samples were randomly selected per group. Different samples (by the group of four) were used in each experiment. Female pups were excluded from this study and allocated to separate analyses of endocrine parameters.

Pregnant dams, initially age-, weight-, and strain-matched, were randomly allocated to either the Control or DEHP-treated groups to ensure even distribution of baseline variability. The male offspring used for the study were also randomly assigned to experimental cages and were numbered by a blinded investigator. Critically, all subsequent behavioral and molecular analyses were conducted by investigators blinded to the prenatal exposure status, thereby ensuring the reliability and unbiased nature of the experimental results.

At eleven weeks of age, animals were euthanized via rapid decapitation using a hand-operated guillotine. This method was selected following the AVMA Guidelines for the Euthanasia of Animals (2020 Edition) (Leary et al., 2020), as it preserves the anatomical integrity of delicate brain regions and prevents chemical interference with downstream analyses. The guillotine was thoroughly cleaned with ethanol and water and wiped dry after each use to avoid cross-contamination. Brains were rapidly extracted on ice, and the hippocampi were dissected for further analysis. Following the brain atlas, the cerebral hemispheres were separated by a midsagittal incision. Using forceps and micro-scissors, the hippocampus was delineated by following the curvature of the alveus and peeled away from the overlying neocortex and underlying thalamic structures. Residual meninges and white matter tracts were carefully removed.

### 2.3 Subcellular fractioning

Subcellular fractions were obtained according to Won et al. (2016). Freshly isolated brain structures were homogenized straight away in a Dounce homogenizer using ice-cold TEVP buffer that contained 320 mM sucrose and 10 mM Tris-HCl (pH 7.5), 5 mM EDTA, 1 mM DTT, 1 mM PMSF, and protease inhibitor cocktail. Every following step was carried out at a temperature of 4°C. The homogenate was centrifuged at 1000 x g for 10 min, followed by centrifugation of the obtained supernatant at 12000 x g for 20 min. Afterwards, the pellet was resuspended in twice the double amount of TEVP (TEV Protease reaction) buffer containing 35.6 mM sucrose, it was incubated for 30 min, and centrifuged at 25,000 x g for 20 min. To obtain the extrasynaptic fraction (not PSD-enriched), the pellet was again resuspended in a triple amount of ice-cold TEVP buffer that included 1 % Triton X-100. Solubilization was carried out for 30 min at 4 °C, following 30 min of centrifugation at 33,000 × g. Then, the supernatant was employed as an "extrasynaptic protein fraction" in the tests. The pellet was resuspended over again in a triple amount of TEVP buffer with 1 % SDS to solubilize the synaptic (PSD-enriched) protein fraction. This was followed by a 2-hour incubation at 37°C with constant, moderate vortexing. Ultimately, the samples underwent a 30-minute centrifugation at 100,000 × g, with the final supernatant being retained and employed as a "synaptic protein fraction." Until further tests, all solubilized proteins were kept in the ultra-low temperature biomedical freezer [Arctiko Ltd. UK] at −80 °C.

### 2.4 Immunoprecipitation

A PTEN antibody [sc-7974, SantaCruz, USA] was incubated with protein A/G agarose in Lysis buffer (pH 7.2) containing 20 mM HEPES, 100 mM NaCl, 1 mM DTT, 1 mM EDTA, 1 mM EGTA, 0.5 % Triton X–100, for 2 h at 4 °C with continuous gentle vortexing at 1:200 dilution per sample. The mixture was then centrifuged for 20 min at 10,000 x g. Samples with equal protein amount (60 µg) were added to the pellet and incubated overnight at 4 °C with constant shaking. After incubation, the samples were centrifuged for 10 min at 1000 x g. 100 mM glycine buffer (pH 3.0) was added to the pellet to dissociate the antigen-antibody connection and incubated on a shaker for three minutes, followed by centrifugation at 10,000 x g for 5 min. 1 M Tris buffer (pH 9.5) was added to the isolated supernatants to neutralize acidity. The final solution was subject to further analysis using the Western blotting method.

### 2.5 Western blotting

The AbcamⓇ Western Blot protocol (*Western Blot Protocol*, 2020) was used to conduct the Western Blotting analysis. Aliquots of the synaptic and extrasynaptic membrane protein fractions (each comprising approximately 20 μg and 30 μg of total protein) were placed onto 4–12 % SDS gels and electrophoresed after being dissolved into equal amounts of protein. Western blotting was performed after the transfer of the isolated proteins onto 0.45 μm nitrocellulose membranes. Ponceau S staining was used to verify proper sample loading and effective protein transfer. Next, the membranes were blocked using 5 % BSA in Tris-buffered saline with 0.1 % Tween-20 (TBST), and then incubated for one hour at room temperature with the following antibodies against the relevant antigens: NHERF [sc-71698, SantaCruz, USA], EAAT2 [ab41621, Abcam, USA], DRD3 [sc-374203, SantaCruz, USA], AMPA [ab31232, Abcam, USA], NMDA λ (GLUN1) [sc-518053, SantaCruz, USA], NMDA ∑1 (GLUN2A) [sc-1468, SantaCruz, USA], NMDA ∑2 (GLUN2B) [sc-1469, SantaCruz, USA], mGlur-5 [sc-47147, SantaCruz, USA], CaMK II [sc13141, SantaCruz, USA], p-CaMK II [sc-32289]). All antibodies were diluted according to the manufacturer's protocols. SantaCruz antibodies (sc-) were diluted in the range 1:1000, Abcam antibodies (ab) dilution range was 1:8000. Following incubation, the membranes were probed for one hour at 20°C using species-appropriate peroxidase-conjugated secondary antibodies and rinsed in TBST. These antibodies were identified by enhanced chemiluminescence autoradiography (ECL kit, sc-2048; Santa-Cruz Biotechnology) following further washing in TBST. Amersham audio graph films were used to expose the blots. The obtained films were digitized and then analyzed with Image Lite Studio software version 5.2.5 (Li-Cor) to quantify the bands' intensities. Western blot analysis was performed according to established protocols, which adhere to current standards for ensuring the accuracy and reliability of quantitative protein detection (Zhang et al., 2025)

### 2.6 Total protein amounts

of the subcellular fractions were determined using a BCA protein assay kit [sc-202389; Santa Cruz Biotechnology, Dallas, TX, USA] according to the manufacturer’s protocol.

### 2.7 PKA (Protein Kinase A) colorimetric activity assay

was performed in the cytosol fraction using the relevant kit [20KA015B, Invitrogen, Thermo Fisher Scientific, Waltham, MA, USA].

### 2.8 Cellular calcineurin phosphatase activity

was measured in cell lysates according to the relevant kit protocol [ab139464, Abcam, Cambridge, UK].

### 2.9 Statistical analysis

Optical density measurements for protein bands were analyzed using one-way analysis of variance (ANOVA). Planned pairwise comparisons between groups were conducted using two-tailed *t*-tests. All statistical analyses were performed using Statistix 9 software (Analytical Software, Tallahassee, FL, USA).

## 3 Results

**Fig. 1:**
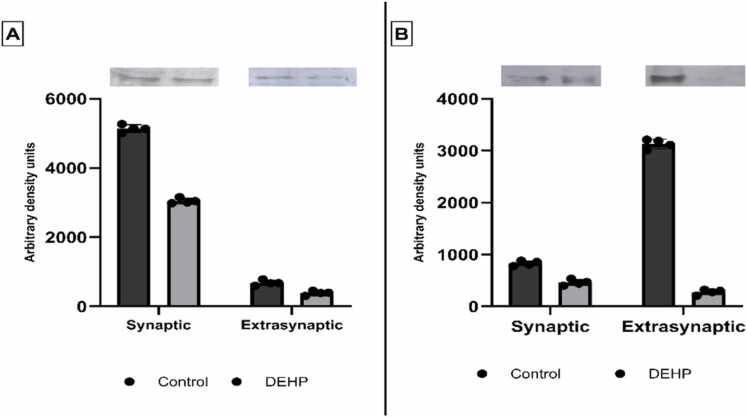
The NHERF1 (A) and the EAAT-2 (B) levels in the PTEN protein interactome in the synaptic and extrasynaptic fractions of the hippocampus of control (n = 4) and DEHP-exposed (n = 4) rats’offspring. In the upper side of the figure are the representative immunoblots illustrating the effect of DEHP on NHERF1 and EAAT-2 expression; The graphs indicate quantification of the effect of DEHP on NHERF and EAAT-2 expression. The intensities of bands were quantified using Image Lite Studio software. Data are normalized and expressed as mean optical density ± standard error of the mean (SEM). (*p < 0.05 versus control).

PTEN is a redox-sensitive lipid and protein phosphatase implicated in cancer and autism spectrum disorders. This protein contains a tandem PDZ domain that modulates the assembly and intracellular trafficking of several transmembrane G protein-coupled receptors and ion transport proteins with the membrane–cytoskeleton adapter proteins (Bhattacharya et al., 2019). Since scaffolding protein NHERF1 influences signaling complex assembly, we first investigated the possible association of PTEN with NHERF1 following phthalate exposure. To analyze the interaction between PTEN and NHERF1, protein complexes from synaptic and extrasynaptic membranes were solubilized and immunoprecipitated using an anti-PTEN antibody. Co-precipitated NHERF1 was subsequently detected via Western blotting. The analysis revealed the presence of PTEN–NHERF1 complexes predominantly in synaptic membrane fractions, with lower levels in extrasynaptic membranes (Fig. 1A). Notably, synaptic PTEN–NHERF1 interactions were markedly diminished in the hippocampus of offspring prenatally exposed to phthalates, indicating disruption of this protein interaction under toxicant influence.

Proteomic characterization of the glutamatergic postsynapse has identified several proteins, including scaffold proteins, glutamate receptors, and ion channels. These protein interactomes show activity-dependent dynamics and stimulus-specific diversity (Lautz et al., 2021). Several types of glutamate receptors in postsynaptic neurons target PTEN through scaffolding proteins such as NHERF1, thereby influencing synaptic activity (Jurado et al., 2010, Rademacher and Eickholt, 2019, Yang et al., 2014). Consistent with this, associations have been identified between PTEN and both glutamate receptors and transporters.

Considering that all known glutamate transporters terminate in a PDZ domain, and this interaction motif is essential in regulating glutamate receptor trafficking and function (Bassan et al., 2008, Song and Huganir, 2002), the association of PTEN with EAAT-2 was next detected. Electrophoretic analysis followed by blotting of PTEN binding proteins unveiled a significant reduction in the expression of the glutamate transporter EAAT-2 in extrasynaptic membrane fractions of offspring following prenatal exposure of parent animals to DEHP (Fig. 1B).

**Fig. 2:**
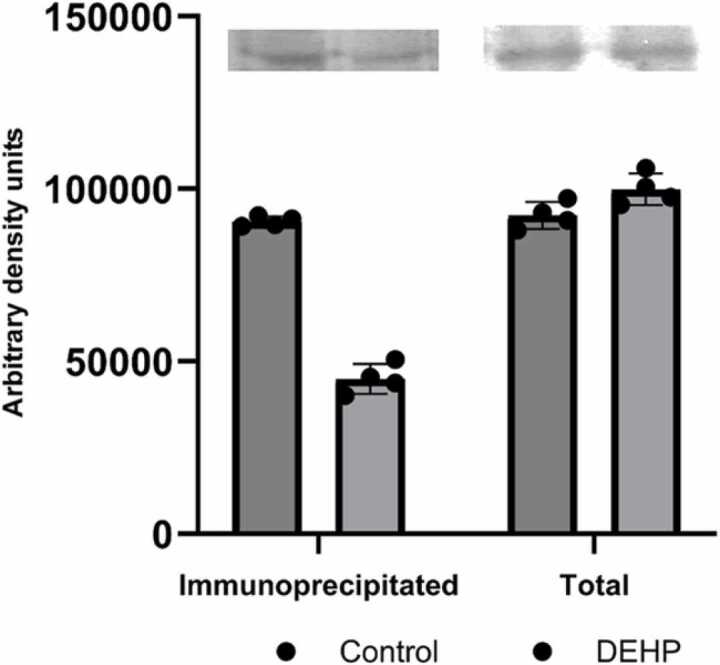
The mGlur 5 total amount and level in the PTEN protein interactome in the synaptic fraction of the hippocampus of control (n = 4) and DEHP-exposed (n = 4) rat offspring. At the top - representative immunoblot illustrating the effect of DEHP on mGlur 5 expression; At the bottom - quantification of the effect of DEHP on mGlur 5 expression. The intensities of bands were quantified using Image Lite Studio software. Data are normalized and expressed as mean optical density ± standard error of the mean (SEM). (*p < 0.05 versus control).

This observation suggests that the DEHP prenatal exposure possibly changes the association of EAAT-2 with PTEN only in non-synaptic regions, which could significantly affect the trafficking and expression of glutamate receptors, particularly mGluR5 and AMPAR (Bodzęta et al., 2021, Moussawi et al., 2011). Further analysis revealed a significant reduction in mGluR5 levels within the PTEN-containing heterocomplex, despite unchanged total mGluR5 expression in the plasma membrane (Fig. 2). This implies that DEHP exposure may interfere with PTEN complex formation, thereby modulating the function of PTEN-associated signaling pathways.

**Fig. 3:**
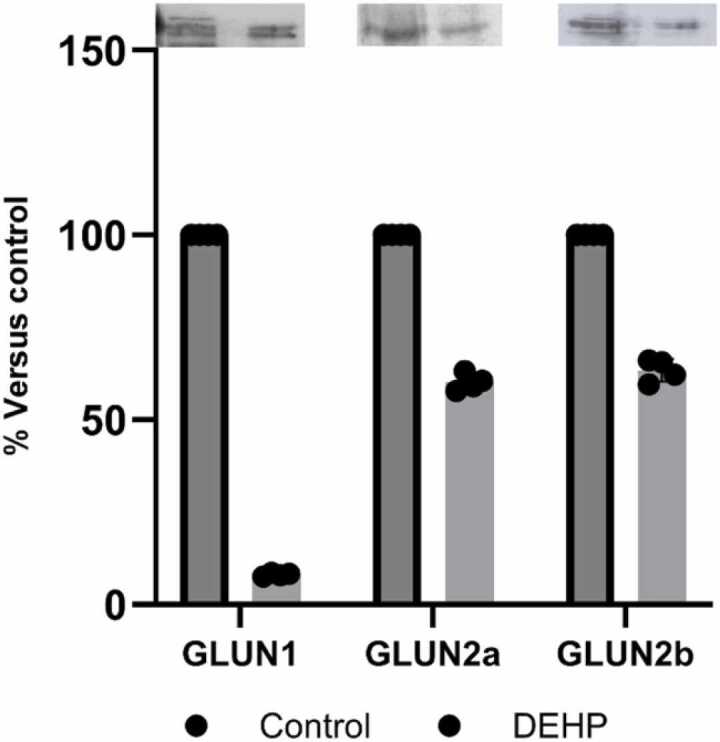
The level of GLUN1, GLUN2a, and GLUN2b in the PTEN protein interactome in the synaptic fraction of the hippocampus of control (n = 4) and DEHP-exposed (n = 4) rat offspring. At the top: representative immunoblots illustrating the effect of DEHP on GLUN1; GLUN2A; GLUN2B expression; At the bottom: quantification of the impact of DEHP on GLUN1; GLUN2A; GLUN2B expression. The intensities of bands were quantified using Image Lite Studio software. Data are normalized and expressed as % versus control ± standard error of the mean (SEM). (*p < 0.05 versus control).

Previous studies have demonstrated that membrane-localized PTEN physically interacts with the GLUN1/GLUN2B complexes of NMDA receptors in hippocampal neurons, leading to the depression of synaptic transmission via NMDA receptor (NMDAR)-dependent long-term depression (LTD) (Jurado et al., 2010). Since PTEN could physically interact with the GLUN1/GLUN2B complex of NMDA receptors in hippocampal neurons, and in a previous study, we have found DEHP-induced changes in different subunits of NMDA receptors (Kiknadze et al., 2025), we have determined the content of NMDAR subunits in the PTEN-associated heterocomplex. The results indicated a sharp reduction in the complexation of all three subunits (GLUN1, GLUN2A, and GLUN2B) with PTEN in phthalate-exposed animal offspring (Fig. 3).

**Fig. 4:**
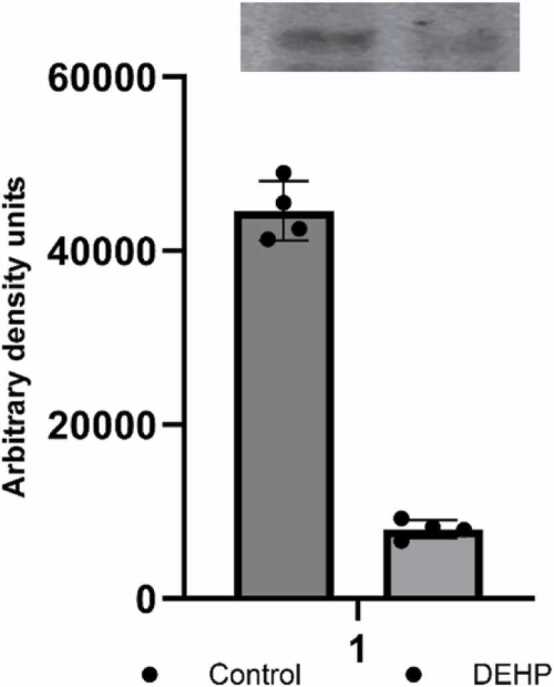
The AMPAR type subunit (GRIA1) level in the PTEN protein interactome in the synaptic fraction of the hippocampus of control (n = 4) and DEHP-exposed (n = 4) rat offspring. At the top - representative immunoblot illustrating the effect of DEHP on GRIA1 expression; At the bottom - quantification of the effect of DEHP on GRIA1 expression. The intensities of bands were quantified using Image Lite Studio software. Data are normalized and expressed as mean optical density ± standard error of the mean (SEM). (*p < 0.05 versus control).

Given that PTEN negatively modulates AMPAR trafficking and surface expression (Moult et al., 2010) PTEN dysfunction may rearrange the location of AMPAR. Subsequently, we have determined the content of the AMPAR type subunit 1 (GRIA1) in the supramolecular complex of PTEN. Co-immunoprecipitation experiments with subsequent Western blotting analysis demonstrated decreased PTEN-bound AMPAR subunit in phthalate-treated animals (Fig. 4). These findings indicate that DEHP possibly alters PTEN's complexation with glutamatergic proteins.

**Fig. 5:**
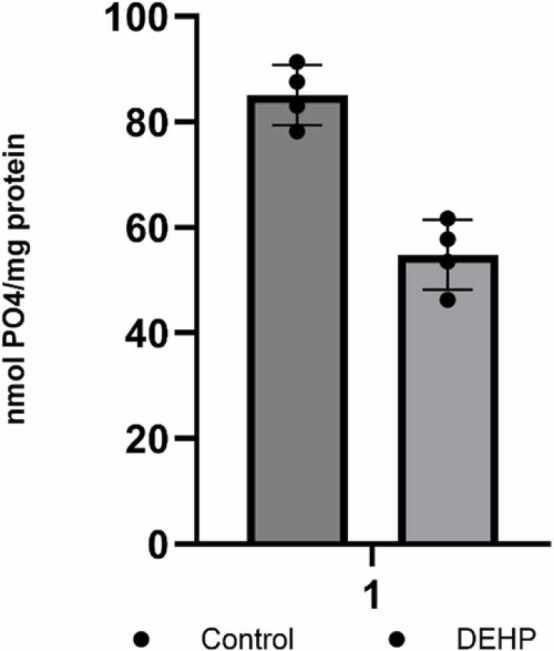
Effect of DEHP prenatal exposure on phosphatase activity of Calcineurin in the hippocampus of control (n = 4) and DEHP-exposed (n = 4) rat offspring. Results are expressed as mean ± SEM. Statistical analysis was carried out by one-way ANOVA *p < 0.05*.*

Thus, prenatal exposure of animals to phthalates induces modifications of the PTEN interactome without affecting the expression of individual proteins. These alterations could be facilitated by phthalate-induced oxidative stress, to which PTEN is particularly susceptible. The oxidation of PTEN may modulate its capacity to interact with binding partners, thereby influencing downstream signaling processes (Verrastro et al., 2016). Alterations in the interactome of proteins are expected to induce changes in the catalytic activity of some downstream effectors and associated proteins. It was shown that PTENα negatively regulates calcineurin activity through direct interaction, and this inhibition could be critical for maintaining proper phosphorylation states of synaptic proteins involved in cognitive processes in the hippocampus (Wang et al., 2017). To elucidate the impact of PPI on the enzymatic activity of calcineurin, we assessed protein phosphatase activity of calcineurin (Fig. 5). Treatment of animals with phthalates resulted in a reduction of calcineurin phosphatase activity, potentially indicating that the masking of the active site of PTEN results in changes in calcineurin activity.

**Fig. 6:**
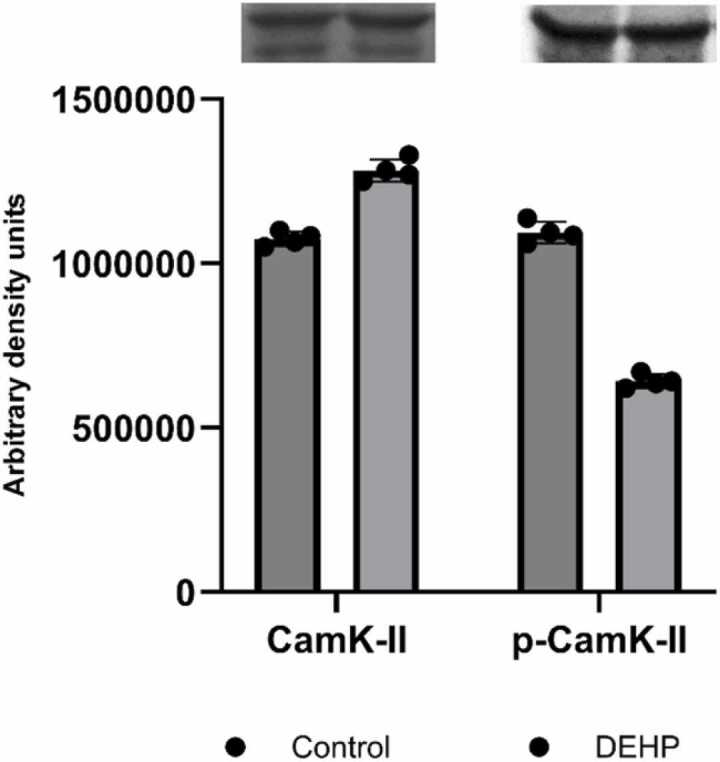
The CamK-II and p-CamK-II levels in the synaptic fraction of the hippocampus of control (n = 4) and DEHP-exposed (n = 4) rat offspring. The intensities of bands were quantified using Image Lite Studio software. Data are normalized and expressed as mean optical density ± standard error of the mean (SEM).

The findings that PTEN can significantly affect calcium-dependent signaling in cells are presented in many literature sources (Pankaew et al., 2022). It was shown that the PTENα isoform directly interacts with and modulates CaMKII, maintaining the kinase in a primed state that enables effective autophosphorylation and long-term potentiation (LTP). By regulating this basal activity of CaMKII, PTENα plays a crucial role in controlling some hippocampal functions (Wang et al., 2017). CaMKII serves as a prominent effector enzyme in calcium signaling in the brain. Its activation is triggered by elevated intracellular calcium levels, leading to the phosphorylation of target proteins involved in various cellular processes, including synaptic vesicle mobilization, gene expression regulation, and modulation of synaptic plasticity. To explore the involvement of this enzyme in the effects of DEHP, we have evaluated the T278 phosphorylation level of CaMKII. Our findings indicate that prenatal exposure to DEHP significantly diminished the autophosphorylation of CaMKII at T278 (Fig. 6).

**Fig. 7:**
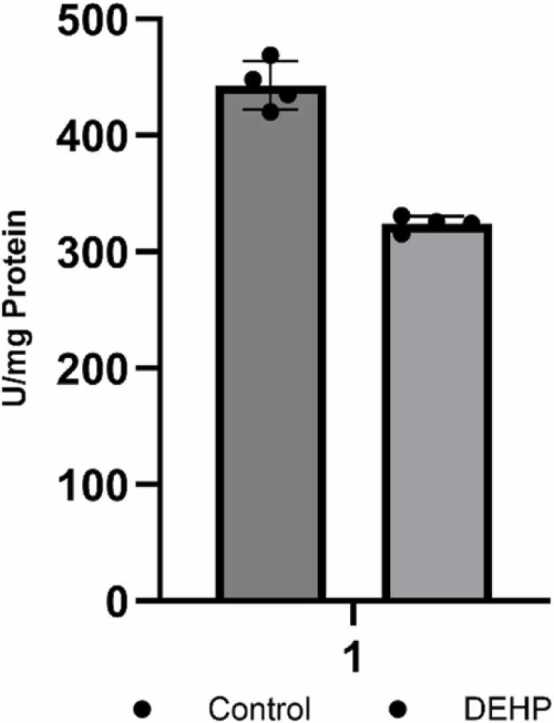
Effect of DEHP prenatal exposure on Protein Kinase A. Results are expressed as mean of units per mg total protein ± SEM (n = 4). Statistical analysis was done by one-way ANOVA (*p < 0.05 versus control).

Since phosphorylation at T278 triggers the translocation of the enzyme into the nucleus, where it facilitates phosphorylation of the cAMP-response element binding protein (CREB) and ultimately alters protein kinase A (PKA) activity, this parameter became the next focus of our investigation. The assessment of PKA activity revealed that prenatal exposure to DEHP results in decreased activity of PKA in the hippocampus of male offspring (Fig. 7), which suggests inhibition of the CAMKII-CREB-dependent pathway.

Therefore, abnormal assembly of the PTEN interactome changes the activity of downstream calcium-dependent regulatory pathways, which can alter synaptic processes in neurons and underlie DEHP-induced neurological impairments.

## 4 Discussion

Phthalates are a series of commonly used chemicals detrimental to human health (Engel et al., 2021). The effects of phthalates on the brain's cognitive functions, learning, memory, and hippocampal processes have been widely studied (Ducroq et al., 2023, Lv et al., 2022, Safarpour et al., 2021). The previous studies suggest that prenatal exposure to low doses of phthalates induces behavioral deficits and exacerbates cellular degenerative processes by initiating oxidative stress (Brassea-Pérez et al., 2022). Analysis of postsynaptic proteins in the hippocampus showed lower glutamatergic neurotransmission in mice exposed to DEHP (Ducroq et al., 2023). Thus, in addition to the effects on steroid hormone metabolism, phthalates could change glutamatergic proteomics (Arkin et al., 2014), which may be associated with various neurodegenerative disorders (Lu et al., 2020).

Being lipophilic, DEHP distributes to the liver, intestines, muscle, kidneys, and fat tissue where it accumulates. Compared to humans, rats metabolize DEHP more rapidly due to the presence of more active esterases and oxidative pathways (Agency for Toxic Substances and Disease Registry (US), 2022, Kurata et al., 2012). In rats, DEHP is rapidly broken down by esterases and lipases into mono(2-ethylhexyl) phthalate (MEHP) and 2-ethylhexanol in the liver and gastrointestinal tract (Choi et al., 2012). In pregnant rats, DEHP and its metabolites cross the placenta and are secreted in milk, exposing fetuses and nursing pups. High lipophilicity and the ability to trigger neuroinflammatory responses also provide the possibility for DEHP to cross the BBB barrier (Ren et al., 2023). Research shows that the mentioned plasticizer penetrates the BBB by increasing its permeability and damaging its normal function. Even in small doses, DEHP can cause neuroinflammation, disrupt neural function, and alter tight junction proteins of the BBB (Ahmadpour et al., 2021). According to different studies, DEHP mostly affects the hippocampus and prefrontal cortex brain structures, although our previous study shows alterations primarily in the hippocampus (Holahan and Smith, 2015, Kang et al., 2021).

Our previous experiments showed that prenatal DEHP exposure results in significant alterations in cognitive behavior and enhanced anxiety of male rat offspring (Kiknadze et al., 2025). Considering that phthalates influence synaptic plasticity, we tested the effect of prenatal exposure of rats to DEHP on PPI in components of glutamatergic synapses in the hippocampus of offspring. Given the elevated sensitivity of regulatory proteins to environmental stress (Smith, 2022), we presume that the effect of phthalates may be associated with the PTEN signaling pathway. PTEN and the downstream signal cascade regulate brain development during cell proliferation and differentiation, migration, neurite outgrowth, synaptogenesis, and myelination. PTEN is prominently expressed in neurons, and recent studies indicate that the dysregulation of its phosphatase activity affects crucial neuronal functions, including synaptic plasticity (Skelton et al., 2020). PTEN loss-of-function leads to progressive neuronal hypertrophy (somatic and dendritic enlargement), hyperconnectivity, and the pathological acceleration of dendritic spine maturation. Crucially, this dysregulation profoundly impacts the cellular mechanisms of memory, causing deficits in both LTP and LTD. This structural and functional imbalance results in an overall state of neuronal hyperexcitability and is directly linked to human neurological disorders such as Autism Spectrum Disorder (ASD), intellectual disability, and epilepsy (Garcia-Junco-Clemente and Golshani, 2014). Protein-protein interaction and the PTEN interactome can influence novel PTEN functions by regulating the complex formation, protein stability, and subcellular localization in various postsynaptic structures, including glutamate receptors, transporters, etc. (Mosessian and Wu, 2010). PTEN's binding to specific PDZ domain-containing proteins enhances PTEN protein stability (Valiente et al., 2005). The multiprotein complex of PTEN can work in an individual regulatory system in a downstream event. Therefore, our study focused on changes in PTEN-associated signaling systems in response to phthalate exposure.

Our results have shown that PTEN forms a heterocomplex with scaffold partners - NHERF in the hippocampus. NHERF modulates the assembly and intracellular trafficking of metabotropic and ionotropic receptors in the glutamatergic synapse (Bodzęta et al., 2021, Filippov et al., 2010) and could directly interact with PTEN (Takahashi et al., 2006). NHERF contains two tandem PDZ domains and a C-terminal sequence that binds several members of the membrane cytoskeletal adapters. Thus, combining these two proteins could form a massive supramolecular scaffold complex capable of binding several other proteins. Based on these results, we have analyzed PTEN interactome proteins in synaptic and extrasynaptic parts of hippocampal membranes. We have found that GLUN1, GLUN2A, and GLUN2B subunits of NMDAR, GRIA1 subunits of AMPAR, mGluR1/5, and EAAT-2 are associated with PTEN, and that the association in male offspring sharply decreased after the prenatal exposure of parent rats to DEHP. Given that the total levels of these glutamatergic synapse proteins remained largely unchanged after phthalate exposure, the observed reduction of these proteins within the heterocomplex is likely due to structural alterations in scaffold proteins such as PTEN and NHERF. Since both scaffold proteins are sensitive to oxidative stress (Smith, 2022, Verrastro et al., 2016) and their modification by ROS changes the protein structure, it can be assumed that oxidative stress in the hippocampal glutamatergic synapses changes protein position in the protein interactome.

The protein redistribution in the PTEN interactome changes the intracellular regulatory effector's activities. Our results have shown that prenatal exposure of rats to DEHP decreased the activities of both calcineurin and CaM kinase II. CaMKII pathway is a central integrator of cellular stress and function. Recent research highlights a novel and specific regulatory role for CaMKII in mediating cellular damage: it acts as a key component of the endoplasmic reticulum ER stress-CaMKII axis that modulates ferroptosis (Zhang et al., 2025). CaMKII pathway represents a critical junction where environmental stressors can convert general cellular toxicity into specific CNS and synaptic dysfunction.

This suppression of calcium-dependent signaling is subsequently accompanied by a reduction in protein kinase A levels in the offspring's hippocampus. CaM-kinase II is predominantly localized in the postsynaptic density of synapses receiving glutamatergic terminals, and the reduction of T286 autophosphorylation impairs learning and memory processes (Zalcman et al., 2018). CaMKII binds to numerous proteins in the post-synaptic density, including NMDA receptors, synapsin 1, F-actin, and calcium channels. The binding and phosphorylation regulate these and other proteins' autonomous activity, location, and transport (Hell, 2014, Lucchesi et al., 2011). Thus, a decrease in autophosphorylation of the enzyme under the influence of phthalates may indicate a decrease in plasticity processes in the hippocampus.

Cyclic AMP/PKA signaling is pivotal for long-term synaptic plasticity and memory types. (Abel and Nguyen, 2008). PKA is coupled directly to NMDARs through an A-kinase anchoring protein, which permits modulation of NMDAR channel activity by PKA (Westphal et al., 1999). PKA-dependent synaptic plasticity links to calcium signaling in spines and the calcium permeability of NMDARs. This mechanism would regulate the amount of calcium influx into hippocampal neurons, a critical determinant of the type of plasticity expressed. PKA-mediated phosphorylation of AMPAR has been implicated in the expression of hippocampal LTP. Serine residues in the intracellular C-terminal domain of Gria1 are phosphorylated by CaMKII and PKA, which is critical to the expression of distinct forms of synaptic plasticity (Abel and Nguyen, 2008). Thus, the downregulation of CaM kinase and PKA activities in offspring after phthalate-treated parent rats affects hippocampal synaptic plasticity and could impair cognitive functions in the brain.

Our present results extend earlier findings on the neurotoxic impact of phthalates on the hippocampus (Kiknadze et al., 2025) by providing new molecular evidence that prenatal DEHP exposure disrupts the composition and functional dynamics of the PTEN protein interactome at excitatory synapses. In our previous study, DEHP exposure was shown to alter PTEN subcellular localization and suppress Akt/mTOR signaling, leading to impaired synaptic transmission and cognitive dysfunction in male offspring. Here, we demonstrate that prenatal DEHP administration also reduces PTEN’s association with key postsynaptic proteins, including the scaffold protein NHERF1, NMDA and AMPA receptor subunits, metabotropic glutamate receptor 5 (mGluR5), and excitatory amino acid transporter-2 (EAAT2), without altering their overall abundance.

By integrating these findings with prior work, we propose that phthalate exposure disrupts hippocampal function through a two-tiered mechanism: first, by destabilizing the PTEN–receptor signaling complex within the postsynaptic density, and second, by inducing a kinase/phosphatase imbalance that alters synaptic responsiveness. Together, these effects may underlie the impaired learning and memory, dendritic abnormalities, and synaptic deficits previously reported after prenatal DEHP exposure (Dong et al., 2021, Kiknadze Natalia et al., 2025)

Therefore, prenatal exposure of rats to DEHP causes changes in the complex formation of these proteins in the interactome, which leads to a decrease in the activity of systems involved in synaptic plasticity in the male offspring. It is assumed that these changes underlie the brain's cognitive function impairment. Further studies are needed to elucidate the role of the PTEN interactome of glutamatergic synapses in the destructive effects of environmental toxins.

## CRediT authorship contribution statement

**Natalia Kiknadze:** Writing – original draft, Software, Investigation, Funding acquisition. **Mikeladze David G:** Writing – review & editing, Funding acquisition, Conceptualization. **Elene Zhuravliova:** Writing – review & editing, Writing – original draft, Visualization, Validation, Project administration, Methodology, Data curation.

## Institutional review board statement

Animal care during experimental procedures was carried out following the recommendations of the Ilia State University Research Projects Ethics Commission and with the Council of Europe Directive 2010/63 / EU on animal experiments.

## Consent for publication

All authors have read and approved the final manuscript for publication.

## Funding

This research was funded by Shota Rustaveli National Science Foundation of Georgia (SRNSFG), grant number: PHDF-21-685

## Conflicts of Interest

The authors declare the following financial interests/personal relationships which may be considered as potential competing interests: David Mikeladze reports financial support and administrative support were provided by Ilia State University. Natalia Kiknadze reports financial support was provided by LEPL Shota Rustaveli National Science Foundation. If there are other authors, they declare that they have no known competing financial interests or personal relationships that could have appeared to influence the work reported in this paper

## References

[bib1] Abel T., Nguyen P.V. (2008). Regulation of hippocampus-dependent memory by cyclic AMP-dependent protein kinase. Prog. Brain Res.

[bib2] Agency for Toxic Substances and Disease Registry (US). 2022. TOXICOKINETICS, SUSCEPTIBLE POPULATIONS, BIOMARKERS, CHEMICAL INTERACTIONS. In Toxicological Profile for Di(2-Ethylhexyl)Phthalate (DEHP). 37040457

[bib3] Ahmadpour D., Mhaouty-Kodja S., Grange-Messent V. (2021). Disruption of the blood-brain barrier and its close environment following adult exposure to low doses of di(2-ethylhexyl)phthalate alone or in an environmental phthalate mixture in male mice. Chemosphere.

[bib4] Arbuckle T.E., Davis K., Boylan K., Fisher M., Fu J. (2016). Bisphenol A, phthalates and lead and learning and behavioral problems in Canadian children 6–11 years of age: CHMS 2007–2009. NeuroToxicology.

[bib5] Arkin M.R., Tang Y., Wells J.A. (2014). Small-molecule inhibitors of protein-protein interactions: progressing toward the reality. Chem. Biol..

[bib6] Bassan M., Liu H., Madsen K.L., Armsen W., Zhou J., DeSilva T., Chen W., Paradise A., Brasch M.A., Staudinger J., Gether U., Irwin N., Rosenberg P.A. (2008). Interaction between the glutamate transporter GLT1b and the synaptic PDZ domain protein PICK1. Eur. J. Neurosci..

[bib7] Baucum A.J. (2017). Proteomic analysis of postsynaptic protein complexes underlying neuronal plasticity. ACS Chem. Neurosci..

[bib8] Bhattacharya S., Stanley C.B., Heller W.T., Friedman P.A., Bu Z. (2019). Dynamic structure of the full-length scaffolding protein NHERF1 influences signaling complex assembly. J. Biol. Chem..

[bib9] Bodzęta A., Scheefhals N., MacGillavry H.D. (2021). Membrane trafficking and positioning of mGluRs at presynaptic and postsynaptic sites of excitatory synapses. Neuropharmacology.

[bib10] Brassea-Pérez E., Hernández-Camacho C.J., Labrada-Martagón V., Vázquez-Medina J.P., Gaxiola-Robles R., Zenteno-Savín T. (2022). Oxidative stress induced by phthalates in mammals: state of the art and potential biomarkers. Environ. Res.

[bib11] Choi K., Joo H., Campbell J.L., Clewell R.A., Andersen M.E., Clewell H.J. (2012). In vitro metabolism of di(2-ethylhexyl) phthalate (DEHP) by various tissues and cytochrome P450s of human and rat. Toxicol. Vitr..

[bib12] Cook C.R., Halden R.U. (2020). Ecological and health issues of plastic waste. Plast. Waste Recycl. Acad. Press.

[bib13] Dong J., Xu X., Zhang Q., Yuan Z., Tan B. (2021). Critical implication of the PTEN/PI3K/AKT pathway during BMP2-induced heterotopic ossification. Mol. Med. Rep..

[bib14] Duarte-Hospital C., Tête A., Brial F., Benoit L., Koual M., Tomkiewicz C., Kim M.J., Blanc E.B., Coumoul X., Bortoli S. (2021). Mitochondrial dysfunction as a hallmark of environmental injury. Cells.

[bib15] Ducroq S., Duplus E., Grange-Messent V., Trivelloni F., Penalva-Mousset L., Petropoulos I., Mhaouty-Kodja S. (2023). Cognitive and hippocampal effects of adult male mice exposure to environmentally relevant doses of phthalates. Environ. Pollut..

[bib16] Dutta S., Haggerty D.K., Rappolee D.A., Ruden D.M. (2020). Phthalate exposure and long-term epigenomic consequences: a review. Front. Genet..

[bib17] Engel S.M., Patisaul H.B., Brody C., Hauser R., Zota A.R., Bennet D.H., Swanson M., Whyatt R.M. (2021). Neurotoxicity of ortho-phthalates: recommendations for critical policy reforms to protect brain development in children. Am. J. Public Health.

[bib18] Filippov A.K., Simon J., Barnard E.A., Brown D.A. (2010). The scaffold protein NHERF2 determines the coupling of P2Y1 nucleotide and mGluR5 glutamate receptor to different ion channels in neurons. J. Neurosci..

[bib19] Frank R.A.W., Komiyama N.H., Ryan T.J., Zhu F., O’Dell T.J., Grant S.G.N. (2016). NMDA receptors are selectively partitioned into complexes and supercomplexes during synapse maturation. Nat. Commun..

[bib20] Garcia-Junco-Clemente P., Golshani P. (2014). PTEN. Commun. Integr. Biol..

[bib21] van Gelder C.A.G.H., Altelaar M. (2021). Neuroproteomics of the synapse: subcellular quantification of protein networks and signaling dynamics. Mol. Cell. Proteom..

[bib22] Hell J.W. (2014). CaMKII: claiming center stage in postsynaptic function and organization. Neuron.

[bib23] Hlisníková H., Petrovičová I., Kolena B., Šidlovská M., Sirotkin A. (2021). Effects and mechanisms of phthalates’ action on neurological processes and neural health: a literature review. Pharmacol. Rep..

[bib24] Holahan M.R., Smith C.A. (2015). Phthalates and neurotoxic effects on hippocampal network plasticity. NeuroToxicology.

[bib25] Hsu P.-C., Jhong J.-Y., Huang L.-P., Lee K.-H., Chen H.-P., Guo Y.-L. (2021). Transgenerational effects of Di(2-Ethylhexyl) phthalate on anogenital distance, sperm functions and DNA methylation in rat offspring. Int. J. Mol. Sci..

[bib26] Huang M.-L., Yen P.-L., Chang C.-H., Liao V.H.-C. (2022). Chronic di(2-ethylhexyl) phthalate exposure leads to dopaminergic neuron degeneration through mitochondrial dysfunction in C. elegans. Environ. Pollut..

[bib27] Huang M.-L., Yen P.-L., Chang C.-H., Liao V.H.-C. (2022). Chronic di(2-ethylhexyl) phthalate exposure leads to dopaminergic neuron degeneration through mitochondrial dysfunction in C. elegans. Environ. Pollut..

[bib28] Jurado S., Benoist M., Lario A., Knafo S., Petrok C.N., Esteban J.A. (2010). PTEN is recruited to the postsynaptic terminal for NMDA receptor-dependent long-term depression. EMBO J..

[bib29] Kang J.S., Baek J.H., Jung S., Chung H.J., Lee D.K., Kim H.J. (2021). Ingestion of Bis(2-ethylhexyl) phthalate (DEHP) during adolescence causes depressive-like behaviors through hypoactive glutamatergic signaling in the medial prefrontal cortex. Environ. Pollut..

[bib30] Kiknadze Natalia, Zhuravliova Elene, Mikeladze David (2025). Prenatal DEHP exposure induces hippocampal neurotoxicity in male offspring via PTEN dysregulation and impaired Akt/mTOR and NMDA signaling. Cell. Mol. Biol..

[bib31] Kougias D.G., Sellinger E.P., Willing J., Juraska J.M. (2018). Perinatal exposure to an environmentally relevant mixture of phthalates results in a lower number of neurons and synapses in the medial prefrontal cortex and decreased cognitive flexibility in adult male and female rats. J. Neurosci..

[bib32] Kurata Y., Makinodan F., Shimamura N., Katoh M. (2012). Metabolism of di (2-ethylhexyl) phthalate (DEHP): comparative study in juvenile and fetal marmosets and rats. J. Toxicol. Sci..

[bib33] Lautz J.D., Tsegay K.B., Zhu Z., Gniffke E.P., Welsh J.P., Smith S.E.P. (2021). Synaptic protein interaction networks encode experience by assuming stimulus-specific and brain-region-specific states. Cell Rep..

[bib34] Steven Leary, Raymond Anthony, Samuel Cartner, Temple Grandin, Cheryl Greenacre, Sharon Gwaltney-Brant, Mary Ann McCrackin, Robert Meyer, David Miller, Jan Shearer, Tracy Turner, & Roy Yanong 2020. AVMA Guidelines for the Euthanasia of Animals: 2020 Edition: Vol. Version 2020.0.1 (2020 Edition).

[bib35] Lu H., Yin K., Su H., Wang D., Zhang Y., Hou L., Li J.B., Wang Y., Xing M. (2023). Polystyrene microplastics induce autophagy and apoptosis in birds lungs via PTEN/PI3K/AKT/mTOR. Environ. Toxicol..

[bib36] Lu H., Zhou Q., He J., Jiang Z., Peng C., Tong R., Shi J. (2020). Recent advances in the development of protein–protein interactions modulators: mechanisms and clinical trials. Signal Transduct. Target. Ther..

[bib37] Lucaccioni L., Trevisani V., Passini E., Righi B., Plessi C., Predieri B., Iughetti L. (2021). Perinatal exposure to phthalates: from endocrine to neurodevelopment effects. Int. J. Mol. Sci..

[bib38] Lucaccioni L., Trevisani V., Passini E., Righi B., Plessi C., Predieri B., Iughetti L. (2021). Perinatal exposure to phthalates: from endocrine to neurodevelopment effects. Int. J. Mol. Sci..

[bib39] Lucchesi W., Mizuno K., Giese K.P. (2011). Novel insights into CaMKII function and regulation during memory formation. Brain Res. Bull..

[bib40] Lupu D.-I., Ulloa A.C., Rüegg J. (2023). Endocrine-disrupting chemicals and hippocampal development: the role of estrogen and androgen signaling. Neuroendocrinology.

[bib41] Lv J., Li Y., Chen J., Li R., Bao C., Ding Z., Ren W., Du Z., Wang S., Huang Y., Wang Q. (2022). Maternal exposure to bis(2-ethylhexyl) phthalate during the thyroid hormone-dependent stage induces persistent emotional and cognitive impairment in middle-aged offspring mice. Food Chem. Toxicol..

[bib42] Ma P., Liu X., Wu J., Yan B., Zhang Y., Lu Y., Wu Y., Liu C., Guo J., Nanberg E., Bornehag C.-G., Yang X. (2015). Cognitive deficits and anxiety induced by diisononyl phthalate in mice and the neuroprotective effects of melatonin. Sci. Rep..

[bib43] Mankidy R., Wiseman S., Ma H., Giesy J.P. (2013). Biological impact of phthalates. Toxicol. Lett..

[bib44] Maric B., Schuster S., Machnik P. (2024). Exposure to phthalate plasticizer compromises normal brain function in an adult vertebrate. Ecotoxicol. Environ. Saf..

[bib45] Matsunaga H., Mizota K., Uchida H., Uchida T., Ueda H. (2010). Endocrine disrupting chemicals bind to a novel receptor, microtubule-associated protein 2, and positively and negatively regulate dendritic outgrowth in hippocampal neurons. J. Neurochem..

[bib46] Mosessian S., Wu H. (2010). PTEN-associated complexes: an overview. Curr. Top. Biochem. Res..

[bib47] Moult P.R., Cross A., Santos S.D., Carvalho A.-L., Lindsay Y., Connolly C.N., Irving A.J., Leslie N.R., Harvey J. (2010). Leptin Regulates AMPA Receptor Trafficking via PTEN Inhibition. J. Neurosci..

[bib48] Moussawi K., Riegel A., Nair S., Kalivas P.W. (2011). Extracellular glutamate: functional compartments operate in different concentration ranges. Front. Syst. Neurosci..

[bib49] Natividade A., Damasceno de Figueiredo N., de Camargo Vieira W., Rodrigues Froes Asmus C.I. (2023). Effects of prenatal exposure to environmental pollutants on birth weight and child weight gain. Curr. Opin. Environ. Sci. Health.

[bib50] Pankaew S., Potier D., Grosjean C., Nozais M., Quessada J., Loosveld M., Remy É., Payet-Bornet D. (2022). Calcium signaling is impaired in PTEN-deficient T cell acute lymphoblastic leukemia. Front. Immunol..

[bib51] Rademacher S., Eickholt B.J. (2019). PTEN in autism and neurodevelopmental disorders. Cold Spring Harb. Perspect. Med..

[bib52] Ren W. qiang, Liu N., Shen Y., Wang X. yan, Zhou Q., Rui C., Yang X. han, Cao S. long, Li L. yu, Wāng Y., Wang Q. nan (2023). Subchronic exposure to di-(2-ethylhexyl) phthalate (DEHP) elicits blood–brain barrier dysfunction and neuroinflammation in male C57BL/6J mice. Toxicology.

[bib53] Rowdhwal S.S.S., Chen J. (2018). Toxic effects of Di-2-ethylhexyl phthalate: an overview. BioMed. Res. Int..

[bib54] RYAN D., MATTHEWS J. (2005). Protein–protein interactions in human disease. Curr. Opin. Struct. Biol..

[bib55] Safarpour S., Ghasemi-Kasman M., Safarpour S., Darban Y.M. (2022). Effects of Di-2-ethylhexyl phthalate on central nervous system functions: a narrative review. Curr. Neuropharmacol..

[bib56] Safarpour S., Zabihi E., Ghasemi-Kasman M., Nosratiyan N., Feizi F. (2021). Prenatal and breastfeeding exposure to low dose of diethylhexyl phthalate induces behavioral deficits and exacerbates oxidative stress in rat hippocampus. Food Chem. Toxicol..

[bib57] Schmidt J.-S., Schaedlich K., Fiandanese N., Pocar P., Fischer B. (2012). Effects of Di(2-ethylhexyl) Phthalate (DEHP) on female fertility and adipogenesis in C3H/N mice. Environ. Health Perspect..

[bib58] Silano V., Barat Baviera J.M., Bolognesi C., Chesson A., Cocconcelli P.S., Crebelli R., Gott D.M., Grob K., Lampi E., Mortensen A., Rivière G., Steffensen I., Tlustos C., Van Loveren H., Vernis L., Zorn H., Cravedi J., Fortes C., Tavares Poças M. de F., Castle L. (2019). Update of the risk assessment of di-butylphthalate (DBP), butyl-benzyl-phthalate (BBP), bis(2-ethylhexyl)phthalate (DEHP), di-isononylphthalate (DINP) and di-isodecylphthalate (DIDP) for use in food contact materials. EFSA J..

[bib59] Skelton P.D., Stan R.V., Luikart B.W. (2020). The role of PTEN in neurodevelopment. Complex Psychiatry.

[bib60] Smith, S.L. 2022. The Biological Role of Redox Signalling by the Tumour Suppressor PTEN. Aston University.

[bib61] Smith S.L., Pitt A.R., Spickett C.M. (2020). Approaches to investigating the protein interactome of PTEN. J. Proteome Res..

[bib62] Song I., Huganir R.L. (2002). Regulation of AMPA receptors during synaptic plasticity. Trends Neurosci..

[bib63] Sree C.G., Buddolla V., Lakshmi B.A., Kim Y.-J. (2023). Phthalate toxicity mechanisms: an update. Compara. Biochem. Physiol. Part C Toxicol. Pharmacol..

[bib64] Takahashi Y., Morales F.C., Kreimann E.L., Georgescu M.-M. (2006). PTEN tumor suppressor associates with NHERF proteins to attenuate PDGF receptor signaling. EMBO J..

[bib65] Tseng I.-L., Yang Y.-F., Yu C.-W., Li W.-H., Liao V.H.-C. (2014). Correction: phthalates induce neurotoxicity affecting locomotor and thermotactic behaviors and AFD neurons through oxidative stress in caenorhabditis elegans. PLoS One.

[bib66] Valiente M., Andrés-Pons A., Gomar B., Torres J., Gil A., Tapparel C., Antonarakis S.E., Pulido R. (2005). Binding of PTEN to specific PDZ domains contributes to PTEN protein stability and phosphorylation by microtubule-associated serine/threonine kinases. J. Biol. Chem..

[bib67] Verrastro I., Tveen-Jensen K., Woscholski R., Spickett C.M., Pitt A.R. (2016). Reversible oxidation of phosphatase and tensin homolog (PTEN) alters its interactions with signaling and regulatory proteins. Free Radic. Biol. Med..

[bib68] Wang P., Mei F., Hu J., Zhu M., Qi H., Chen X., Li R., McNutt M.A., Yin Y. (2017). PTENα modulates CaMKII signaling and controls contextual fear memory and spatial learning. Cell Rep..

[bib69] Wang P., Mei F., Hu J., Zhu M., Qi H., Chen X., Li R., McNutt M.A., Yin Y. (2017). PTENα modulates CaMKII signaling and controls contextual fear memory and spatial learning. Cell Rep..

[bib70] Wang Y., Qian H. (2021). Phthalates and their impacts on human health. Healthcare.

[bib71] Westphal R.S., Tavalin S.J., Lin J.W., Alto N.M., Fraser I.D.C., Langeberg L.K., Sheng M., Scott J.D. (1999). Regulation of NMDA receptors by an associated phosphatase-kinase signaling complex. Science.

[bib72] Won S., Incontro S., Nicoll R.A., Roche K.W. (2016). PSD-95 stabilizes NMDA receptors by inducing the degradation of STEP 61. Proc. Natl. Acad. Sci..

[bib73] Xu X., Yang Y., Wang R., Wang Y., Ruan Q., Lu Y. (2015). Perinatal exposure to di-(2-ethylhexyl) phthalate affects anxiety- and depression-like behaviors in mice. Chemosphere.

[bib74] Yang D.-J., Wang X.-L., Ismail A., Ashman C.J., Valori C.F., Wang G., Gao S., Higginbottom A., Ince P.G., Azzouz M., Xu J., Shaw P.J., Ning K. (2014). PTEN regulates AMPA receptor-mediated cell viability in iPS-derived motor neurons. Cell Death Dis..

[bib75] Zalcman G., Federman N., Romano A. (2018). CaMKII Isoforms in learning and memory: localization and function. Front. Mol. Neurosci..

[bib76] Zhang Y., Hou L., Guo T., Lu H., Zhang X., Xing M. (2025). An in-depth analysis of the effects of excessive acetochlor exposure on chicken liver health. Pestic. Biochem. Physiol..

[bib77] Zhang Y., Lu H., Hou L., Zhang X., Guo T., Wang R., Wang Q., Xing M. (2025). GPR120 exacerbates the immune-inflammatory response in chicken liver by mediating acetochlor induced macrophage M1 polarization. J. Hazard. Mater..

[bib78] Zhu F., Cizeron M., Qiu Z., Benavides-Piccione R., Kopanitsa M.V., Skene N.G., Koniaris B., DeFelipe J., Fransén E., Komiyama N.H., Grant S.G.N. (2018). Architecture of the mouse brain synaptome. Neuron.

